# Synthesis, Characterization, and Biological Activity Studies on Fanlizhicyclopeptide A

**Published:** 2017

**Authors:** Rajiv Dahiya, Sunil Singh

**Affiliations:** a *Laboratory of Peptide Research and Development,* *School of Pharmacy, Faculty of Medical Sciences, The University of the West Indies, St. Augustine, Trinidad & Tobago, West Indies.*; b *Research Scholar, Department of Pharmacy, Mewar University, Gangrar, Chittorgarh, Rajasthan, India.*

**Keywords:** Fanlizhicyclopeptide A, Cyclic heptapeptide, Solution-Phase synthesis, Macrocyclization, Pharmacological activity

## Abstract

The synthesis of a proline-rich cyclic heptapeptide, fanlizhicyclopeptide A (8), previously isolated from the fruits of Annona squamosa (sugar-apples), is described via coupling of tetrapeptide -prolyl--tyrosyl--leucyl--proline methyl ester with tripeptide Boc-glycyl--valyl--proline followed by cyclization of the linear fragment having seven amino acid units. Structure of the synthesized cyclooligopeptide was confirmed by the means of chemical and spectroscopic methods including FTIR, ^1^H NMR, ^13^C NMR, FABMS and further, subjected to the anthelmintic, antibacterial and the antifungal activity studies. Bioactivity results indicated that the newly synthesized cyclic peptide displayed potent anthelmintic activity against the three earthworm species* Megascoplex konkanensis*, *Pontoscotex corethruses* and *Eudrilus eugeniae *at 2 mg/mL and remarkable anti-dermatophytic activities against *Trichophyton mentagrophytes *and *Microsporum audouinii* at concentration of 6 *µ*g/mL.

## Introduction

Ever since ancient times, in search for rescue for their disease, the people looked for drugs in nature. Many of the powerful drugs were used in modern medicines originated in plants. Herbal medicines have a strong traditional or conceptual base and the potential to be useful as drugs in terms of safety and effectiveness leads to treating different diseases (1). Today’s plant-based drugs treat a range of diseases, from headaches to cancer. Awareness of medicinal plants usage is a result of the many years of struggles against illnesses due to which man learned to pursue drugs in barks, seeds, fruit bodies, and other parts of the plants (2). Nowadays, cyclooligopeptides from plant fruits have received more attention of researchers and scientists (3-6) because of their complex molecular structures and a wide spectrum of associated pharmacological activities like anti-inflammatory activity (7), vasorelaxant activity (8), cell growth inhibitory activity (9), and immunosuppressive activity (10). A natural cyclic heptapeptide, fanlizhicyclopeptide A was isolated from the fruits of Annona squamosa (sugar-apples) and its structure was elucidated by ESI MS/MS, 1D and 2D NMR data, and chemical degradation (11). 

Keeping in view broad range of pharmacological activities possessed by cyclooligopeptides (12-15) and to obtain a natural cyclic peptide in good yield in the laboratory, the present study aimed toward the solution-phase synthesis, structure elucidation and the screening of a proline-rich cycloheptapeptide, fanlizhicyclopeptide A for the anthelmintic, antibacterial and antifungal potential.

## Experimental

*Chemistry*

Melting point was determined by the open capillary method and is uncorrected. IR spectra were recorded using an FTIR-8400S fourier transform spectrophotometer (Shimadzu, Kyoto, Japan). ^1^H NMR and ^13^C NMR spectra were recorded on a Bruker AC 300 spectrometer at 300 MHz (Brucker, IL, USA). Mass spectra were recorded on a JMS-DX 303 spectrometer (Jeol, Tokyo, Japan). Elemental analysis was performed on a Vario EL III elemental analyzer (Elementar Vario EL III, Hanau, Germany) and optical rotation of the synthesized peptides was measured on an Optics Technology automatic polarimeter (OpticsTech, Delhi, India). Purity of the synthesized peptides was checked by TLC on precoated silica gel G plates (Kieselgel 0.25 mm, 60G F_254_, Merck, Germany). 

*General method for the synthesis of linear tri/tetrapeptide segments (5, 6)*

To the solution of the amino acid methyl ester hydrochloride or dipeptide methyl ester (0.01 mol) in dichloromethane (DCM, 20 mL), NMM (2.23 mL, 0.021 mol) was added at 0 °C, and the reaction mixture was stirred for 15 min. The Boc-dipeptide (0.01 mol) in DCM (20mL),*N*-(3-Dimethylaminopropyl)-*N′*-ethylcarbodiimide  hydrochloride (EDC.HCl, 1.92 g, 0.01 mol), and HOBt (1.34 g, 0.01 mol) were added with stirring to the above reaction mixture. Stirring of the resulting mixture was continued for 24 h at r.t. The reaction mixture was filtered and the residue was washed with DCM (20 mL) and added to the filtrate. The filtrate was washed with 5% NaHCO_3 _and saturated NaCl solutions. The organic layer was dried over anhydrous Na_2_SO_4_, filtered, and evaporated in vacuum. The crude product was recrystallized from a mixture of chloroform and petroleum ether (b.p. 40-60 °C) followed by cooling at 0 °C to get the title compounds. 

*tert-Butyloxycarbonyl-* *l* *-Prolyl-L-Tyrosyl-* *l* *-Leucyl-* *l* *-Proline methyl ester (5)*

Semisolid mass; Yield 89%; [*α*]_D_ = –76.3 ° (*c* = 0.25, MeOH); R_f_ = 0.69 (CHCl_3_·MeOH - 8:2); IR (CHCl_3_):* v *= 3373 (O–H_str_, aromatic ring), 3127, 3124 (N–H_str_, amide), 3069-3062 (Ar−H_str_, aromatic ring), 2999-2992 (C–H_str_, cyclic CH_2_), 2968, 2924-2921 (C–H_str_, asym, CH_3_ and CH_2_), 2843, 2839 (C–H_str_, sym, CH_2_), 1743 (C=O_str_, ester), 1669-1666, 1643, 1639 (C=O_str_, 3° and 2° amide), 1565, 1437 (skeletal bands), 1534, 1530 (N–H_def_, amide), 1388, 1369 (C–H_def_, tert-butyl), 1383, 1362 (C–H_def_, iso-propyl), 1271 (C–O_str_, ester), 716, 689 (C−H_def_, oop, aromatic ring) cm^−1^; ^1^H NMR (CDCl_3_): *δ* = 7.52 (br. s, 1 H, N*H*, Leu), 6.99, 6.96 (dd, *J* = 8.55, 5.25 Hz, 2 H, *m*-H’s, Tyr), 6.92 (br. s, 1 H, N*H*, Tyr), 6.89, 6.86 (dd, *J* = 8.6, 4.9 Hz, 2 H, *o*-H’s, Tyr), 5.95 (br. s, 1 H, O*H*, Tyr), 4.56-4.52 (q, *J* = 7.9 Hz, 1 H, *α*-H, Tyr), 4.15-4.11 (m, *J* = 6.7 Hz, 1 H, *α*-H, Leu), 4.10 (t, *J* = 6.9 Hz, 1 H, *α*-H, Pro-1), 3.89 (t, *J* = 6.85 Hz, 1 H, *α*-H, Pro-2), 3.62 (s, 3 H, OC*H*_3_), 3.39 (t, *J* = 7.15 Hz, 2 H, *δ*-H, Pro-2), 3.23 (t, *J* = 7.2 Hz, 2 H, *δ*-H, Pro-1), 2.89 (d, *J* = 5.5 Hz, 2 H, *β*-H’s, Tyr), 2.57-2.54 (m, 2 H, *β*-H’s, Pro-1), 2.03-1.98 (m, 4 H, *β*-H’s, *γ*-H’s, Pro-2), 1.93-1.89 (m, 2 H, *γ*-H’s, Pro-1), 1.76 (t, 2 H, *J* = 5.9 Hz, *β*-H’s, Leu), 1.49 (s, 9 H, tert-butyl), 1.47-1.42 (m, 1 H, *γ*-H, Leu), 0.98 (d, 6 H, *J* = 6.25 Hz, *δ*-H’s, Leu); C_31_H_46_N_4_O_8 _(602): calcd. C 61.78, H 7.69, N 9.30; found C 61.75, H 7.68, N 9.32.

*tert-Butyloxycarbonyl-Glycyl-* *l* *-Valyl-* *l* *-Proline methyl ester* *(6)*

Semisolid mass; Yield 79%; [*α*]_D_ = +49.2 ° (*c* = 0.25, MeOH); R_f_ = 0.56 (CHCl_3_·MeOH - 8:2); IR (CHCl_3_):* v *= 3128, 3123 (N–H_str_, amide), 2999-2993 (C–H_str_, cyclic CH_2_), 2925-2921 (C–H_str_, asym, CH_2_), 2853, 2849 (C–H_str_, sym, CH_2_), 1743 (C=O_str_, ester), 1667, 1645 (C=O_str_, 3° and 2° amide), 1537 (N–H_def_, 2° amide), 1389, 1368 (C–H_def_, tert-butyl), 1382, 1363 (C–H_def_, iso-propyl), 1268 (C–O_str_, ester) cm^−1^; ^1^H NMR (CDCl_3_): *δ* = 7.83 (br. s, 1 H, N*H*, Val), 6.82 (br. s, 1 H, N*H*, Gly), 4.45 (t, *J* = 5.85 Hz, 1 H, *α*-H, Val), 3.75 (d, *J* = 5.5 Hz, 2 H, *α*-H’s, Gly), 3.65 (t, 1 H, *J* = 6.9 Hz, *α*-H, Pro), 3.63 (s, 3 H, OC*H*_3_), 3.12 (t, 2 H, *J* = 7.15 Hz, *δ*-H, Pro), 2.06-1.97 (m, 5 H, *β*-H’s, *γ*-H’s, Pro and *β*-H, Val), 1.54 (s, 9 H, tert-butyl), 1.07 (d, 6 H, *J* = 4.6 Hz, *γ*-H’s, Val); C_18_H_31_N_3_O_6 _(385): calcd. C 56.09, H 8.11, N 10.90; found C 56.11, H 8.14, N 10.89. 

*Deprotection of the tetrapeptide unit at the amino terminal*

Boc-protected tetrapeptide 5 (6.02 g, 0.01 mol) was dissolved in CHCl_3_ (15 mL) and treated with CF_3_COOH (2.28 g, 0.02 mol). The resulting solution was stirred at room temperature for 1 h and washed with a saturated NaHCO_3_ solution (25 mL). The organic layer was dried over anhydrous Na_2_SO_4_ and concentrated under reduced pressure. The crude product was purified by crystallization from CHCl_3_ and petroleum ether (b.p. 40-60 °C) to get the pure deprotected compound 5a. 

**Table 1:** Anthelmintic evaluation data for the linear and cycloheptapeptide 7, 8.

	**Earthworm species**					
	**M. konk.**		**P. core.**		**E. euge.**	
	**Mean paralyzing time (min)** ‡	**Mean** **death** **time (min)** ‡	**Mean paralyzing time (min)**	**Mean** **death** **time (min)**	**Mean paralyzing time (min)**	**Mean** **death** **time (min)**
7	14.35 ± 0.13	23.09 ± 0.31	18.04 ± 0.40	28.49 ± 0.17	13.22 ± 0.11	24.55 ± 0.47
8	10.08 ± 0.42	17.13 ± 0.49	13.66 ± 0.27	22.08 ± 0.21	11.28 ± 0.34	20.18 ± 0.22
Control#	–	–	–	–	–	–
Mebendazole	13.63 ± 0.33	22.43 ± 0.27	17.56 ± 0.49	29.49 ± 0.15	13.50 ± 0.39	24.07 ± 0.44

M. konk.: Megascoplex konkanensis, P. core.: Pontoscotex corethruses, E. euge.: Eudrilus eugeniae

‡ Data are given as mean ± S.D. (n = 3)

# Tween 80 (0.5%) in distilled water

**Table 2:** Antimicrobial evaluation data for linear and cycloheptapeptide 7, 8

**Compound**	**Diameter of zone of inhibition (mm)**							
	**Bacterial strains**				**Fungal strains**			
	**B.** **sub.**	**S.** **aur.**	**P.** **aeru.**	**K.** **pneu.**	**C.** **alb.**	**M.** **audo.**	**A.** **niger**	**T.** **menta.**
7	–	–	14(12.5)	–	10(25)	17(6)	–	18(6)
8	11(25)	–	17(12.5)	14(25)	13(25)	21(6)	10(25)	22(6)
Control*	–	–	–	–	–	–	–	–
Gatifloxacin	18(12.5)†	27(6)	23(6)	25(6)	–	–	–	–
Griseofulvin	–	–	–	–	20(6)	18(6)	20(12.5)	19(6)

B. sub.: Bacillus subtilus, S. aur.: Staphylococcus aureus, P. aeru.: Pseudomonas aeruginosa, K. pneu.: Klebsiella pneumoniae, C. alb.: Candida albicans, M. audo.: Microsporum audouinii,

A. niger: Aspergillus niger, T. menta.: Trichophyon mentagrophytes

† Values in bracket are MIC values (g/mL)

* DMF / DMSO

**Scheme 1:**
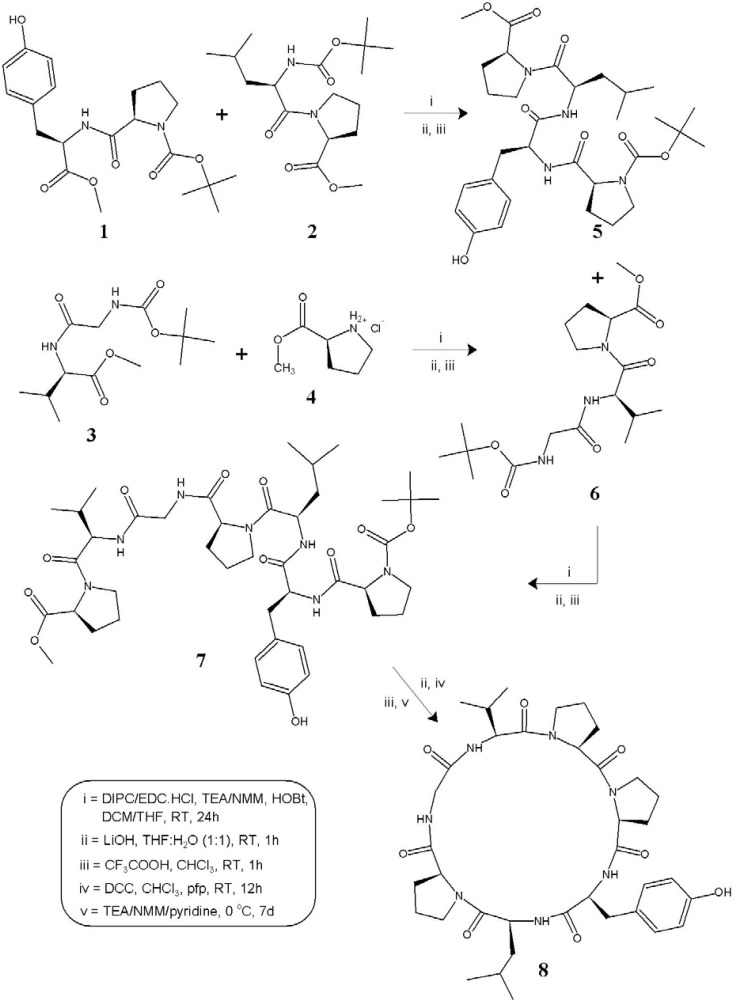
Synthetic route to the cycloheptapeptide (fanlizhicyclopeptide A) 8.

*L-Prolyl-L-Tyrosyl-L-Leucyl-L-Proline methyl ester (5a)*

Semisolid mass; Yield 76%; [*α*]_D_ = –43.8 ° (*c* = 0.25, MeOH). R_f_ = 0.75 (CHCl_3_·MeOH - 8:2); IR (CHCl_3_):* v *= 3369 (N–H_str_, amine), 3371 (O–H_str_, aromatic ring), 3129, 3126 (N–H_str_, amide), 3067-3062 (Ar−H_str_, aromatic ring), 2997-2991 (C–H_str_, cyclic CH_2_), 2969, 2926, 2920 (C–H_str_, asym, CH_3_ and CH_2_), 2845, 2841 (C–H_str_, sym, CH_2_), 1741 (C=O_str_, ester), 1667, 1642-1638 (C=O_str_, 3° and 2° amide), 1617 (N–H_def_, amine), 1565, 1436 (skeletal bands), 1536, 1529 (N–H_def_, amide), 1382, 1361 (C–H_def_, iso-propyl), 1273 (C–O_str_, ester), 715, 687 (C−H_def_, oop, aromatic ring) cm^−1^; ^1^H NMR (CDCl_3_): *δ* = 7.95 (br. s, 1 H, N*H*, Tyr), 7.49 (br. s, 1 H, N*H*, Leu), 6.98, 6.95 (dd, *J* = 8.6, 5.25 Hz, 2 H, *m*-H’s, Tyr), 6.91, 6.87 (dd, *J* = 8.55, 4.85 Hz, 2 H, *o*-H’s, Tyr), 6.44 (br. s, 2 H, O*H*, Tyr and N*H*, Pro-1), 4.36-4.31 (q, *J* = 7.85 Hz, 1 H, *α*-H, Tyr), 4.14-4.09 (m, *J* = 6.65 Hz, 1 H, *α*-H, Leu), 3.92 (t, *J* = 6.9 Hz, 1 H, *α*-H, Pro-2), 3.64 (s, 3 H, OC*H*_3_), 3.50 (t, *J* = 6.85 Hz, 1 H, *α*-H, Pro-1), 3.42 (t, *J* = 7.2 Hz, 2 H, *δ*-H, Pro-2), 2.95 (d, *J* = 5.45 Hz, 2 H, *β*-H’s, Tyr), 2.76 (t, *J* = 7.15 Hz, 2 H, *δ*-H, Pro-1), 2.06-1.99 (m, 4 H, *β*-H’s, -H’s, Pro-2), 1.89-1.85 (m, 2 H, *β*-H’s, Pro-1), 1.78-1.73 (m, 2 H, *γ*-H’s, Pro-1), 1.67 (t, 2 H, *J* = 5.85 Hz, *β*-H’s, Leu), 1.52-1.47 (m, 1 H, *γ*-H, Leu), 0.99 (d, 6 H, *J* = 6.3 Hz, *δ*-H’s, Leu); C_26_H_38_N_4_O_6 _(502): calcd. C 62.13, H 7.62, N 11.15; found C 62.15, H 7.65, N 11.12.

*Deprotection of the tripeptide unit at the carboxyl terminal*

To a solution of the tripeptide 6 (3.85 g, 0.01 mol) in THF·H_2_O (1:1, 36 mL), LiOH (0.36 g, 0.015 mol) was added at 0 °C. The mixture was stirred at room temperature for 1 h and then acidified to pH 3.5 with 1 H_2_SO_4_. The aqueous layer was extracted with Et_2_O (3 × 25 mL). The combined organic extracts were dried over anhydrous Na_2_SO_4_ and concentrated under reduced pressure. The crude product was finally crystallized from methanol and ether to get the pure deprotected compound 6a.

*tert-Butyloxycarbonyl-Glycyl-* *l* *-Valyl-* *l* *-Proline* *(6a)*

Semisolid mass; Yield 88%; [*α*]_D_ = +114.7° (*c* = 0.25, MeOH); R_f_ = 0.79 (CHCl_3_·MeOH - 8:2); IR (CHCl_3_):* v *= 3295-2517 (O–H_str_, CO*OH*), 3129, 3122 (N–H_str_, amide), 2997-2991 (C–H_str_, cyclic CH_2_), 2927, 2922 (C–H_str_, asym, CH_2_), 2852, 2849 (C–H_str_, sym, CH_2_), 1715 (C=O_str_, *CO*OH), 1665, 1642 (C=O_str_, 3° and 2° amide), 1535 (N–H_def_, 2° amide), 1387, 1369 (C–H_def_, tert-butyl), 1384, 1362 (C–H_def_, iso-propyl) cm^−1^; ^1^H NMR (CDCl_3_): *δ* = 10.46 (br. s, 1 H, O*H*, COO*H*), 7.82 (br. s, 1 H, N*H*, Val), 6.85 (br. s, 1 H, N*H*, Gly), 5.25 (t, *J* = 5.9 Hz, 1 H, *α*-H, Val), 3.82 (t, 1 H, *J* = 6.85 Hz, *α*-H, Pro), 3.74 (d, *J* = 5.45 Hz, 2 H, *α*-H’s, Gly), 3.13 (t, 2 H, *J* = 7.2 Hz, *δ*-H, Pro), 2.07-1.98 (m, 5 H, *β*-H’s, *γ*-H’s, Pro and *β*-H, Val), 1.55 (s, 9 H, tert-butyl), 1.05 (d, 6 H, *J* = 4.55 Hz, *γ*-H’s, Val); C_17_H_29_N_3_O_6 _(371): calcd. C 54.97, H 7.87, N 11.31; found C 54.95, H 7.88, N 11.33. 

*Procedure for preparation of linear heptapeptide unit and its cyclized form (7, 8)*

Tetrapeptide methyl ester, -Pro--Tyr--Leu--Pro-OMe (5a, 5.02 g, 0.01 mol) was dissolved in 30 mL of THF and 2.8 mL (0.021 mol) of TEA was added at 0 °C and the resulting mixture was stirred for 15 min. Boc-protected tripeptide, Boc-Gly--Val--Pro-OH (6a, 3.71 g, 0.01 mol) was dissolved in 30 mL of THF and DIPC/EDC.HCl (1.26 g/1.92 g, 0.01 mol) and HOBt (1.34 g, 0.01 mol) were added to above mixture with stirring. Stirring was continued for 24 h, after which the reaction mixture was filtered and the filtrate was washed with 25 mL each of 5% NaHCO_3 _and saturated NaCl solutions. The organic layer was dried over anhydrous Na_2_SO_4_, filtered and evaporated in vacuum. The crude product was recrystallized from a mixture of chloroform and petroleum ether (b.p. 40-60 °C) followed by cooling at 0 °C to get the Boc-Gly--Val--Pro--Pro--Tyr--Leu--Pro-OMe (7) as yellowish semisolid mass. Linear heptapeptide unit (7, 4.28 g, 0.005 mol) was then, deprotected at the carboxyl terminal using lithium hydroxide (LiOH, 0.18 g, 0.0075 mol) to obtain the Boc-Gly--Val--Pro--Pro--Tyr--Leu--Pro-OH. To a solution of the deprotected heptapeptide (4.21 g, 0.005 mol) in CHCl_3 _(50 mL), pentafluorophenol (*pfp*, 1.23 g, 0.0067 mol) and DCC (1.06 g, 0.005 mol) were added followed by stirring at r.t. for 12 h. Filtrate of the above reaction mixture was washed with 10% NaHCO_3_ (3 × 20 mL) and 5% HCl (2 × 20 mL) solutions to obtain the corresponding pentafluorophenyl ester Boc-Gly--Val--Pro--Pro--Tyr--Leu--Pro-O*pfp*. Boc-group of the resulting unit (4.03 g, 0.004 mol) was removed using CF_3_COOH (0.91 g, 0.008 mol) to get the deprotected product Gly--Val--Pro--Pro--Tyr--Leu--Pro-O*pfp *which was dissolved in CHCl_3 _(25 mL) and TEA/NMM/pyridine (2.8 mL/2.21mL/1.61 mL, 0.021 mol) was added. Then, the whole contents were kept at 0 °C for 7 days. The reaction mixture was washed with 10% NaHCO_3_ (3 × 25 mL) and 5% HCl (2 × 25 mL) solutions. The organic layer was dried over anhydrous Na_2_SO_4 _and crude cyclized compound was recrystallized from CH_2_Cl_2_/*n*-hexane to obtain the pure product *cyclo *(Glycyl--Valyl--Prolyl--Prolyl--Tyrosyl--Leucyl--Proyl) (8).

*tert-Butyloxycarbonyl-Glycyl-L-Valyl-L-Prolyl-L-Prolyl-L-Tyrosyl-L-Leucyl-L-Proline.(7)*

Semisolid mass; Yield 69%; [*α*]_D_ = –63.4 ° (*c* = 0.25, MeOH); R_f_ = 0.49 (CHCl_3_·MeOH - 9:1); IR (CHCl_3_):* v *= 3374 (O–H_str_, aromatic ring), 3129-3125, 3122 (N–H_str_, amide), 3064-3059 (Ar−H_str_, aromatic ring), 2999-2992 (C–H_str_, cyclic CH_2_), 2969, 2926-2918 (C–H_str_, asym, CH_3_ and CH_2_), 2851-2843 (C–H_str_, sym, CH_2_), 1742 (C=O_str_, ester), 1669-1665, 1644, 1639 (C=O_str_, 3° and 2° amide), 1568, 1435 (skeletal bands), 1537, 1533-1528 (N–H_def_, 2° amide), 1389, 1372 (C–H_def_, tert-butyl), 1382, 1363 (C–H_def_, iso-propyl), 1271 (C–O_str_, ester), 712, 689 (C−H_def_, oop, aromatic ring) cm^−1^; ^1^H NMR (CDCl_3_): *δ* = 7.84 (br. s, 1 H, N*H*, Val), 7.52 (br. s, 1 H, N*H*, Leu), 6.99, 6.96 (dd, *J* = 8.55, 5.3 Hz, 2 H, *m*-H’s, Tyr), 6.90, 6.86 (dd, *J* = 8.6, 4.9 Hz, 2 H, *o*-H’s, Tyr), 6.79 (br. s, 1 H, N*H*, Gly), 6.51 (br. s, 1 H, N*H*, Tyr), 5.95 (br. s, 1 H, O*H*, Tyr), 4.83-4.79 (q, *J* = 7.9 Hz, 1 H, *α*-H, Tyr), 4.48 (t, *J* = 5.85 Hz, 1 H, *α*-H, Val), 4.42 (t, 1 H, *J* = 6.9 Hz, *α*-H, Pro-2), 4.13-4.08 (m, *J* = 6.7 Hz, 1 H, *α*-H, Leu), 3.93 (t, 1 H, *J* = 6.85 Hz, *α*-H, Pro-3), 3.88 (t, *J* = 6.9 Hz, 1 H, *α*-H, Pro-1), 3.72 (d, *J* = 5.5 Hz, 2 H, *α*-H’s, Gly), 3.68 (t, 2 H, *J* = 7.15 Hz, *δ*-H, Pro-2), 3.62 (s, 3 H, OC*H*_3_), 3.41 (t, *J* = 7.2 Hz, 2 H, *δ*-H, Pro-3), 3.08 (t, *J* = 7.15 Hz, 2 H, *δ*-H, Pro-1), 2.89 (d, *J* = 5.5 Hz, 2 H, *β*-H’s, Tyr), 2.71-2.67 (m, 2 H, *β*-H’s, Pro-1), 2.65-2.61 (m, 2 H, *β*-H’s, Pro-2), 2.07-1.99 (m, 5 H, *β*-H’s, *γ*-H’s, Pro-3 and *β*-H, Val), 1.95-1.89 (m, 4 H, *γ*-H’s, Pro-2 and Pro-1), 1.82 (t, 2 H, *J* = 5.9 Hz, *β*-H’s, Leu), 1.53 (s, 9 H, tert-butyl), 1.50-1.45 (m, 1 H, *γ*-H, Leu), 1.08 (d, 6 H, *J* = 4.6 Hz, *γ*-H’s, Val), 0.98 (d, 6 H, *J* = 6.3 Hz, *δ*-H’s, Leu); ^13^C NMR (CDCl_3_): *δ* = 174.2 (C=O, Tyr), 172.5, 169.8, 168.4 (3 C, C=O, Pro-2, Pro-1 and Pro-3), 167.9, 167.1 (2 C, C=O, Leu and Val), 165.6 (C=O, Gly), 157.7 (C=O, Boc), 152.2 (*p*-C, Tyr), 130.7 (2 C, *o*-C’s, Tyr), 129.3 (*γ*-C, Tyr), 128.0 (2 C, *m*-C’s, Tyr), 79.3 (*α*-C, Boc), 60.5, 58.6, 55.9 (3 C, *α*-C’s, Pro-1, Pro-3 and Pro-2), 52.9 (O*C*H_3_), 49.4 (*α*-C, Val), 48.6, 47.8 (2 C, *α*-C’s, Leu and Tyr), 47.5, 46.0, 44.7 (3 C, -C’s, Pro-1, Pro-2 and Pro-3), 42.0 (*α*-C, Gly), 39.6, 37.0 (2 C, *β*-C’s, Leu and Tyr), 31.5, 29.2 (2 C, *β*-C’s, Pro-1 and Val), 28.9 (*β*-C, Pro-3), 26.7 (3 C, *β*-C’s, Boc), 25.5 (*β*-C, Pro-2), 24.9, 24.5, 23.8 (3 C, *γ*-C’s, Pro-3, Pro-1 and Pro-2), 22.0 (*γ*-C, Leu), 21.4 (2 C, *δ*-C’s, Leu), 18.7 (2 C, *γ*-C’s, Val); C_43_H_65_N_7_O_11 _(856): calcd. C 60.33, H 7.65, N 11.45; found C 60.35, H 7.66, N 11.47. 

*(Glycyl-L-Valyl-L-Prolyl-L-Prolyl-L-Tyrosyl-L-Leucyl-LProlyl) (8)*

Pale yellow solid; m.p. 137-139 °C (d); Yield 85 % (C_5_H_5_N), 78 % (NMM), 68 % (TEA); [*α*]_D_ = –74.2° (*c* = 0.54, MeOH) (–74.1° for natural fanlizhicyclopeptide A [11]); R_f_ = 0.77 (CHCl_3_·MeOH - 9:1); IR (KBr):* v *= 3372 (O–H_str_, aromatic ring), 3128-3125, 3123-3119 (N–H_str_, amide), 3067-3061 (ArH_str_, aromatic ring), 2999, 2996-2991 (C–H_str_, cyclic CH_2_), 2967, 2925-2919 (C–H_str_, asym, CH_3_ and CH_2_), 2853, 2949-2843 (C–H_str_, sym, CH_2_), 1668-1664, 1642, 1639 (C=O_str_, 3° and 2° amide), 1566, 1439 (skeletal bands), 1538, 1532-1529 (N–H_def_, 2 amide), 1380, 1362 (C–H_def_, iso-propyl), 716, 687 (C−H_def_, oop, aromatic ring) cm^−1^; ^1^H NMR (CDCl_3_): *δ* = 9.88 (br. s, 1 H, N*H*, Tyr), 9.69 (br. s, 1 H, N*H*, Leu), 9.18 (br. s, 1 H, N*H*, Gly), 7.85 (br. s, 1 H, N*H*, Val), 6.99, 6.95 (dd, *J* = 8.6, 5.25 Hz, 2 H, *m*-H’s, Tyr), 6.89, 6.86 (dd, *J* = 8.55, 4.9 Hz, 2 H, *o*-H’s, Tyr), 6.55 (t, *J* = 5.9 Hz, 1 H, *α*-H, Val), 6.33-6.28 (m, *J* = 6.7 Hz, 1 H, *α*-H, Leu), 5.97 (br. s, 1 H, O*H*, Tyr), 4.25 (t, 1 H, *J* = 6.85 Hz, *α*-H, Pro-2), 4.21-4.37 (q, *J* = 7.85 Hz, 1 H, *α*-H, Tyr), 4.02 (d, *J* = 5.45 Hz, 2 H, *α*-H’s, Gly), 3.89 (t, 1 H, *J* = 6.9 Hz, *α*-H, Pro-3), 3.75 (t, *J* = 6.85 Hz, 1 H, *α*-H, Pro-1), 3.51 (t, 2 H, *J* = 7.2 Hz, *δ*-H, Pro-2), 3.23 (t, *J* = 7.15 Hz, 2 H, *δ*-H, Pro-3), 2.95 (t, *J* = 7.2 Hz, 2 H, *δ*-H, Pro-1), 2.71-2.65 (m, 4 H, *β*-H’s, Pro-1 and Pro-3), 2.64-2.60 (m, 2 H, *β*-H’s, Pro-2), 2.57 (d, *J* = 5.45 Hz, 2 H, *β*-H’s, Tyr), 1.89 (t, 2 H, *J* = 5.85 Hz, *β*-H’s, Leu), 1.87-1.78 (m, 6 H, *γ*-H’s, Pro-2, Pro-3 and Pro-1), 1.67-1.62 (m, 1 H, *β*-H, Val), 1.15 (d, 6 H, *J* = 4.55 Hz, *γ*-H’s, Val), 0.99 (d, 6 H, *J* = 6.25 Hz, *δ*-H’s, Leu), 0.86-0.79 (m, 1 H, *γ*-H, Leu); ^13^C NMR (CDCl_3_): *δ* = 173.9 (C=O, Leu), 172.0, 171.3 (2 C, C=O, Pro-3 and Tyr), 170.8, 170.3, (2 C, C=O, Pro-2 and Val), 169.7, 169.1 (2 C, C=O, Pro-1 and Gly), 153.8 (*p*-C, Tyr), 135.6 (*γ*-C, Tyr), 131.3 (2 C, *m*-C’s, Tyr), 129.1 (2 C, *o*-C’s, Tyr), 62.2, 58.4, 56.1 (3 C, *α*-C’s, Pro-3, Pro-2 and Pro-1), 55.8 (*α*-C, Val), 54.9, 54.5 (2 C, *α*-C’s, Leu and Tyr), 49.3, 46.5, 45.1 (3 C, *δ*-C’s, Pro-3, Pro-2 and Pro-1), 43.6, 42.0 (2 C, *β*-C’s, Leu and Tyr), 40.9 (*α*-C, Gly), 34.6, 33.7, 30.1 (3 C, *β*-C’s, Pro-3, Pro-1 and Pro-2), 29.9 (*β*-C, Val), 29.0 (*γ*-C, Leu), 24.1, 23.8 (2 C, *γ*-C’s, Pro-3 and Pro-1), 23.1 (2 C, *δ*-C’s, Leu), 20.7 (*γ*-C, Pro-2), 18.9 (2 C, *γ*-C’s, Val); MS (FAB, 70 eV): *m/z* (%) = 724 (100) [M + 1]^+^, 696 (11) [724-CO]^+^, 667 (39) [Val-Pro-Pro-Tyr-Leu-Pro]^+^, 639 (17) [667-CO]^+^, 627 (78) [Pro-Tyr-Leu-Pro-Gly-Val]^+^, 625 (49) [Pro-Pro-Tyr-Leu-Pro-Gly]^+^, 611 (64) [Pro-Gly-Val-Pro-Pro-Tyr]^+^, 599 (19) [627-CO]^+^, 597 (16) [625-CO]^+^, 583 (16) [611-CO]^+^, 570 (48) [Val-Pro-Pro-Tyr-Leu]^+^, 568 (37) [Pro-Pro-Tyr-Leu-Pro]^+^, 542 (11) [570-CO]^+^, 540 (13) [568-CO]^+^, 530 (76) [Tyr-Leu-Pro-Gly-Val]^+^, 528 (41) [Pro-Tyr-Leu-Pro-Gly]^+^, 502 (11) [530-CO]^+^, 500 (15) [528-CO]^+^, 471 (76) [Pro-Pro-Tyr-Leu]^+^, 457 (23) [Val-Pro-Pro-Tyr]^+^, 448 (52) [Pro-Gly-Val-Pro-Pro]^+^, 443 (29) [471-CO]^+^, 431 (23) [Tyr-Leu-Pro-Gly]^+^, 429 (16) [457-CO]^+^, 420 (11) [448-CO]^+^, 403 (14) [431-CO]^+^, 374 (48) [Pro-Tyr-Leu]^+^, 358 (61) [Pro-Pro-Tyr]^+^, 351 (72) [Pro-Gly-Val-Pro]^+^, 346 (17) [374-CO]^+^, 330 (14) [358-CO]^+^, 323 (16) [351-CO]^+^, 294 (38) [Val-Pro-Pro]^+^, 277 (41) [Tyr-Leu]^+^, 261 (33) [Pro-Tyr]^+^, 254 (33) [Pro-Gly-Val]^+^, 233 (10) [261-CO]^+^, 226 (14) [254-CO]^+^, 195 (27) [Pro-Pro]^+^, 167 (11) [195-CO]^+^, 155 (29) [Pro-Gly]^+^, 136 (19) [Tyr immonium ion, C_8_H_10_NO]^+^, 127 (21) [155-CO]^+^, 107 (10) [C_7_H_7_O]^+^, 98 (22) [Pro]^+^, 93 (13) [C_6_H_5_O]^+^, 86 (21) [Leu immonium ion, C_5_H_12_N]^+^, 72 (26) [Val immonium ion, C_4_H_10_N]^+^, 70 (34) [Pro immonium ion, C_4_H_8_N]^+^, 57 (14) [C_4_H_9_]^+^, 43 (28) [C_3_H_7_]^+^, 30 (16) [Gly immonium ion, CH_4_N]^+^, 17 (10) [OH]^+^, 15 (21) [CH_3_]^+^; C_37_H_53_N_7_O_8 _(723): calcd. C 61.39, H 7.38, N 13.54; found C 61.41, H 7.36, N 13.55.

*Anthelmintic evaluation*

Newly synthesized linear heptapeptide and heptacyclopeptide 7, 8 were subjected to anthelmintic activity studies against three earthworm species *Megascoplex konkanensis*, *Pontoscotex corethruses* and *Eudrilus eugeniae* at 2 mg/mL concentration using Garg’s method (16). Tween 80 (0.5%) in distilled water was used as control and mebendazole was used as standard drug. The results of anthelmintic screening are compiled in Table 1.

*Antibacterial and antifungal evaluation*

The synthesized linear and heptacyclopeptide 7, 8 were evaluated for their antimicrobial activity against Gram-positive bacteria *Bacillus subtilis*, *Staphylococcus aureus*, Gram-negative bacteria *Pseudomonas aeruginosa*, *Klebsiella pneumoniae*, dermatophytes *Microsporum audouinii*, *Trichophyton mentagrophytes*, diamorphic fungi *Candida albicans,* and other fungal strains, including *Aspergillus niger* at 25−6 *μ*g/mL concentration by using modified Kirby-Bauer disc diffusion method (17). MIC values of test compounds were determined by tube dilution technique. The Petri plates inoculated with bacterial cultures were incubated at 37 °C for 18 h and those inoculated with fungal cultures were incubated at 37 °C for 48 h. Gatifloxacin and griseofulvin were used as reference drugs and DMF/DMSO were used as control. The results of antibacterial and antifungal studies are presented in Table 2. Experimental details of the biological activity studies are described in our previously published reports (18-22).

## Results and Discussion

*Chemistry*

The cycloheptapeptide molecule was split into three dipeptide units Boc--Pro--Tyr-OMe (1), Boc--Leu--Pro-OMe (2), and Boc-Gly--Val-OMe (3) and also a single amino acid unit -Pro-OMe·HCl (4). Dipeptide units 1-3 were prepared by coupling of Boc-amino acids such as Boc--Pro, Boc--Leu, and Boc-Gly with corresponding amino acid methyl ester hydrochlorides such as -Tyr-OMe·HCl, -Pro-OMe·HCl, and -Val-OMe·HCl by following the modified Bodanzsky and Bodanzsky method (23). After deprotection at the carboxy terminus, dipeptide 1 was coupled with dipeptide 2 deprotected at the amino terminus, to get the tetrapeptide unit Boc--Pro--Tyr--Leu--Pro-OMe (5). The carboxyl group of dipeptide 3 was removed by alkaline hydrolysis using lithium hydroxide (LiOH) and the deprotected peptide was coupled with amino acid unit 4, utilizing three different carbodiimides to get the tripeptide unit Boc-Gly--Val--Pro-OMe (6). After removal of the ester group of tripeptide 6 and the Boc group of tetrapeptide 5, the deprotected units were coupled to get the linear heptapeptide unit Boc--Pro--Tyr--Leu--Pro-Gly--Val--Pro-OMe (7). The methyl ester group of the linear peptide fragment was replaced by pentafluorophenyl (*pfp*) ester group. The Boc-group of resulting compound was removed using trifluoroacetic acid (CF_3_COOH), and the deprotected linear fragment was now cyclized by keeping the whole contents at 0 °C for 7 days in the presence of catalytic amounts of TEA or NMM or pyridine to get the cyclic product 8 (Scheme 1). 

The structure of the newly synthesized cyclooligopeptide as well as that of the intermediate tri/tetra/heptapeptides was confirmed by FT-IR, ^1^H NMR spectroscopy and elemental analysis. In addition, mass spectra and ^13^C NMR spectroscopy were recorded for the linear and cyclic heptapeptide.

The synthesis of cyclooligopeptide 8 was accomplished with 85% yield, and pyridine proved to be an effective base for cyclization of the linear heptapeptide unit. Cyclization of the linear peptide fragment was supported by the disappearance of absorption bands at 1742, 1271 and 1387, 1373 cm^−1 ^(C=O_str_, C–O_str_, ester and C–H_def_, tert-butyl groups) in IR spectra of 8. The formation of the cyclopeptide was further confirmed by the disappearance of singlets at 3.62 and 1.53 ppm corresponding to three protons of the methyl ester group and nine protons of the tert-butyl group of Boc in the ^1^H NMR spectrum and the disappearance of the singlets at 157.7, 79.3 and 52.9, 26.7 ppm corresponding to carbon atoms of ester and tert-butyl groups in the ^13^C NMR spectrum of 8. Furthermore, the ^1^H NMR and ^13^C NMR spectra of the synthesized cyclic heptapeptide showed characteristic peaks confirming the presence of all the 53 protons and 37 carbon atoms. The appearance of the pseudomolecular ion peak (M + 1)^+^ at *m/z* = 724 corresponding to the molecular formula C_37_H_53_N_7_O_8 _in the mass spectrum of 8, along with other fragment ion peaks resulting from cleavage at ‘Pro-Val’, ‘Pro-Pro’, ‘Pro-Leu’, ‘Tyr-Pro’ and ‘Val-Gly’ amide bonds showed the exact sequence of the attachment of all the seven amino acid moieties in a chain. In addition, the presence of the immonium ion peaks at *m/z* = 136 (Tyr), 86 (Leu), 72 (Val), 70 (Pro), and 30 (Gly) further confirmed all the amino acid moieties in the cyclopeptide structure. Furthermore, the elemental analysis of 8 afforded values with tolerance of ± 0.02 strictly in accordance with the molecular composition. 

*Biological activity*

Comparison of antifungal activity data suggested that 8 possessed potent bioactivity against dermatophytes* M. audouinii *and* T. mentagrophytes* with MIC values of 6 *μ*g/mL when compared to the reference drug griseofulvin. From the analysis of anthelmintic activity data, it is observed that 8 displayed remarkable activity against all three earthworm species* M. konkanensis*, *P. corethruses* and *E. eugeniae*, in comparison to standard drug mebendazole. Moreover, a moderate level of activity was observed against Gram-negative bacteria *P. aeruginosa* for the newly synthesized cyclopeptide, in comparison to the standard drug gatifloxacin. However, 8 displayed no significant activity against neither Gram-positive and Gram-negative bacteria nor *Aspergillus niger*. In addition, the analysis of the pharmacological activity data revealed that heptacyclopeptide 8 displayed a higher bioactivity against pathogenic fungi and earthworms than its linear form 7, which is due to the fact that cyclization of peptides reduces the degree of freedom for each constituent within the ring and thus substantially leads to reduced flexibility, increased potency, and selectivity of cyclic peptides.

## Conclusion

In conclusion, a first total successful synthesis of the natural peptide fanlizhicyclopeptide A (8) was accomplished in the present study, with good yield via coupling reactions utilizing different carbodiimides. The DIPC / TEA coupling method proved to be yield-effectice, in comparison to methods utilizing EDC·HCl / DIPC and NMM, providing 10-12% additional yield. The pentafluorophenyl ester was shown to be better for the activation of the acid functionality of the linear heptapeptide unit. Pyridine was found to be a good base for the intramolecular cyclization of the linear peptide fragment in comparison to TEA or NMM. The synthesized heptacyclopeptide displayed potent anthelmintic activity and effectiveness against pathogenic dermatophtytes. In addition, Gram-negative bacteria were found to be more sensitive than Gram-positive bacteria towards the newly synthesized peptide. On passing toxicity tests, heptacyclopeptide 8 may prove as a good candidate for clinical studies and can be a new anti-dermatophyte and anthelmintic drug of future.
